# Demographic and Lifestyle Characteristics, but Not Apolipoprotein E Genotype, Are Associated with Intelligence among Young Chinese College Students

**DOI:** 10.1371/journal.pone.0143157

**Published:** 2015-11-17

**Authors:** Xiao-Fen Chen, Zichen Wei, Tingting Wang, Zhen-Lian Zhang, Yiwei Wang, Michael G. Heckman, Nancy N. Diehl, Yun-Wu Zhang, Huaxi Xu, Guojun Bu

**Affiliations:** 1 Fujian Provincial Key Laboratory of Neurodegenerative Disease and Aging Research, Institute of Neuroscience, Medical College, Xiamen University, Xiamen, 361102, China; 2 The first affiliated hospital of Xiamen university, Xiamen, 361003, China; 3 Division of Biomedical Statistics and Informatics, Mayo Clinic, Jacksonville, FL, 32224, United States of America; 4 Department of Neuroscience, Mayo Clinic, Jacksonville, FL, 32224, United States of America; Second Affiliated Hospital, Zhejiang University, CHINA

## Abstract

**Background:**

Intelligence is an important human feature that strongly affects many life outcomes, including health, life-span, income, educational and occupational attainments. People at all ages differ in their intelligence but the origins of these differences are much debated. A variety of environmental and genetic factors have been reported to be associated with individual intelligence, yet their nature and contribution to intelligence differences have been controversial.

**Objective:**

To investigate the contribution of apolipoprotein E (*APOE*) genotype, which is associated with the risk for Alzheimer’s disease, as well as demographic and lifestyle characteristics, to the variation in intelligence.

**Methods:**

A total of 607 Chinese college students aged 18 to 25 years old were included in this prospective observational study. The Chinese revision of Wechsler Adult Intelligence Scale (the fourth edition, short version) was used to determine the intelligence level of participants. Demographic and lifestyle characteristics data were obtained from self-administered questionnaires.

**Results:**

No significant association was found between *APOE* polymorphic alleles and different intelligence quotient (IQ) measures. Interestingly, a portion of demographic and lifestyle characteristics, including age, smoking and sleep quality were significantly associated with different IQ measures.

**Conclusions:**

Our findings indicate that demographic features and lifestyle characteristics, but not *APOE* genotype, are associated with intelligence measures among young Chinese college students. Thus, although *APOE* ε4 allele is a strong genetic risk factor for Alzheimer’s disease, it does not seem to impact intelligence at young ages.

## Introduction

Intelligence is the ability to learn from experience and to learn and adapt to changing environment. It predicts a wide range of social outcomes. People with higher intelligence tend to perform better in educational attainment, income, health and longevity [1]. Individuals differ from one another in their intellectual abilities, thus understanding the genetic and environmental risk factors that contribute to individual differences is of great interest to a wide range of researchers and to the general population. There are numerous studies reporting the associations between a biological variable and individual intelligence; however, findings of these studies are typically difficult to replicate [1–3]. Behavioral studies using twin-, family- and adoption-based designs suggest that a proportion of the population variance in intelligence was attributed to environmental and genetic differences [3, 4]. There are many different environmental influences that have been found to shape intelligence, including nutrition, education, pollution, drug and alcohol abuse [5–8]. However, no single gene or gene variant has been identified that is robustly associated with individual intelligence. An emerging finding was that older people with the ε4 allele of the gene for apolipoprotein E (*APOE*), a strong genetic risk factor for Alzheimer’s disease (AD), are more likely to have faster cognitive decline [9, 10].

ApoE is a major cholesterol carrier and plays an important role in maintaining brain lipid homeostasis [11]. The human *APOE* gene exists as three polymorphic alleles—ε2 (Cys112, Cys158), ε3 (Cys112, Arg158), and ε4 (Arg112, Arg158)—which have a worldwide frequency of 8.4%, 77.9% and 13.7%, respectively [12]. *APOE* polymorphic alleles are the main genetic determinants of AD risk [13, 14]. The ε4 allele of *APOE* dramatically increases the risk of developing AD in a gene dose-dependent manner. The mean age of onset and frequency of AD are 68 years and 91% in ε4 homozygotes, 76 years and 47% in ε4 heterozygotes, 84 years and 20% in ε4 non-carriers [15]. Conversely, the ε2 allele of *APOE* has a protective effect against developing AD [12]. ApoE isoforms differentially regulate amyloid-β (Aβ) aggregation and clearance in the brain [16–18], and have distinct functions in regulating brain lipid transport [19], glucose metabolism [20], neuronal signaling [21], neuroinflammation [22], and mitochondrial function [23, 24]. Mechanistically, apoE isoforms appear to modulate the risk of AD through both Aβ-dependent and Aβ-independent pathways [25]. In addition to increasing the risk for AD, *APOE* ε4 allele also accelerates cognitive decline in elderly non-demented individuals [9, 10]. However, whether *APOE* genotype influences cognitive function or related measures at early ages is less clear.

Studying the influence of *APOE* alleles on cognition at an early age is of particular interest as it would potentially aid mechanistic understanding of the function of apoE; however, there have been relatively few studies examining the association of *APOE* polymorphic alleles with cognitive function in young adults. Within the few existing literature, there remain large discrepancies concerning the effect of *APOE* genotype on cognitive performance in early life [26–29]. Therefore, in this study we sought to examine the associations between *APOE* genotype and intelligence-test scores among Chinese college students aged 18 to 25 years old. We also examined the associations of demographic and lifestyle characteristics with intelligence-test scores to further understand the mechanism underlying individual intelligence differences.

## Materials and Methods

Approval for this study was obtained from the ethics committees of Xiamen University and AnFang College. All individuals who participated in this study gave written informed consent.

### Study population

A total of 607 Chinese college students aged 18 to 25 years old from Xiamen University (N = 433) or AnFang College (N = 174) in the southeast city of Xiamen, Fujian Province were included in this prospective observational study. In addition to intelligence-test scores (intelligence quotient, IQ), demographic and lifestyle characteristics data were obtained from self-administered questionnaires. Demographic information was collected regarding age, gender, height, weight, and body mass index (BMI). Lifestyle factors that were collected included personality (introvert or extrovert), smoking (No or Yes), alcohol consumption (No or Yes), physical exercise (Often, once a week, once a month, or rare), and sleep quality (High, Intermediate, or Low).

### Intelligence tests

The Chinese revision of Wechsler Adult Intelligence Scale (the fourth edition, short version) was used to determine the intelligence level of participants. The scores are summed into four indexes (Verbal Comprehension Index or VCI, Working Memory Index or WMI, Processing Speed Index or PSI, and Perceptual Reasoning Index or PRI) and one Full Scale IQ which ranges from the lowest 72 to the highest 152 points.

### *APOE* genotyping and quality control

Genomic DNA was extracted from saliva samples using a DNA extraction kit (Zeesan Biotech, Xiamen). Genotyping of the two *APOE* SNPs (rs429358:T/C and rs7412:C/T) was carried out using the *APOE* SNP genotyping kits (Memorigen Biotech, Xiamen, China) and the Applied Biosystems^®^ 7500 Real-Time PCR Systems (Applied Biosystems, Foster City, CA). Data analysis was performed by measuring the allele-specific fluorescence. The genotyping analysis had been repeated at least twice, and was blind to different investigators. Additionally, three samples with known *APOE* genotypes were included in each assay. Finally, 10% of the total samples were randomly selected and retested with 100% concordance.

### Statistical analysis

Continuous variables were summarized with the sample median, minimum, first quartile, third quartile, and maximum. Due to the small number of students with an ε4/ε4 genotype (N = 7), we utilized a dominant model (i.e. presence vs. absence of the ε4 allele) in all analyses where the primary interest was *APOE* ε4. Similarly, since only 4 students had an ɛ2/ɛ2 genotype, a dominant model was used in all analyses where the focus was *APOE* ɛ2. We examined the associations of *APOE* ε4 allele with Full Scale IQ and each of the four indexes using single variable (i.e. unadjusted) and multivariable linear regression models. Multivariable models were adjusted for school (Xiamen University or AnFang College), age, gender, height, weight, BMI, personality, smoking, drinking, physical exercise, and sleep quality in order to account for the potential confounding influences of these variables. Associations between *APOE* ε2 and IQ measures were evaluated in a similar manner.

Associations of demographic and lifestyle characteristics with Full Scale IQ and each of the four indexes were also evaluated using single variable and multivariable linear regression models, where multivariable models were adjusted for age, gender, height, weight, BMI, personality, smoking, drinking, physical exercise, and sleep quality. Regression coefficients and 95% confidence intervals (CIs) were estimated in all linear regression analysis, where regression coefficients are interpreted as the change in the mean IQ measure corresponding to the specified increase (continuous variables) or presence of the given characteristic (categorical variables). In order to adjust for multiple testing, we utilized a Bonferroni correction separately for each group of similar tests. Specifically, p-values of 0.01 or lower were considered as statistically significant when evaluating the associations of *APOE* ε4 or ε2 with IQ measures (5 tests of association were performed for ε4 or ε2), while p-values of 0.005 or lower were considered as significant when examining associations of demographic and lifestyle characteristics with IQ measures (10 tests of association were performed for each IQ measure). All statistical analysis was performed using SAS (version 9.2; SAS Institute, Inc., Cary, North Carolina).

## Results

### Subject description

As shown in Table 1, median age was 20 (Range: 18–25), 263 subjects (43.3%) were male, and median BMI was 20.0 (Range: 14.5–35.5). The majority of subjects were extroverts (67.1%), do not smoke (81.7%), or drink alcohol (72.3%). The amount of physical exercise was most commonly often (32.9%) or once a week (32.3%), and sleep quality was either intermediate (60.5%) or high (27.4%) for most subjects. Median Full Scale IQ score was 112 (Range: 72–152), median VCI was 110 (Range: 65–140), median WMI was 110 (Range: 60–140), median PSI was 110 (Range: 55–145), and median PRI was 110 (Range: 65–130). *APOE* genotype distributions were ε2/ε2 for 4 students (0.7%), ε2/ε3 for 72 students (11.9%), ε2/ε4 for 12 students (2.0%), ε3/ε3 for 412 students (67.9%), ε3/ε4 for 100 students (16.5%), and ε4/ε4 for 7 students (1.2%). Therefore, allele frequencies for ɛ2, ɛ3 and ɛ4 were 7.6%, 82.0%, 10.4%, respectively.

**Table 1:** Student characteristics and IQ information.

Variable	Summary (N = 607)
Age	20 (18, 19, 22, 25)
Gender (Male)	263 (43.3%)
Height (m)	1.66 (1.48, 1.60, 1.73, 1.93)
Weight (kg)	55 (34, 50, 62, 98)
BMI	20.0 (14.5, 18.7, 21.7, 35.5)
*APOE* genotype	
ε2/ε2	4 (0.7%)
ε2/ε3	72 (11.9%)
e2/e4	12 (2.0%)
ε3/ε3	412 (67.9%)
ε3/ε4	100 (16.5%)
ε4/ε4	7 (1.2%)
*APOE* allele frequency	
ɛ2	7.6%
ɛ3	82.0%
ɛ4	10.4%
Personality	
Extrovert	381 (67.1%)
Introvert	187 (32.9%)
Smoking	
No	470 (81.7%)
Yes	105 (18.3%)
Alcohol consumption	
No	159 (27.7%)
Yes	416 (72.3%)
Physical exercise	
Often	189 (32.9%)
Once a week	186 (32.3%)
Once a month	59 (10.3%)
Rare	141 (24.5%)
Sleep quality	
High	157 (27.4%)
Intermediate	347 (60.5%)
Low	70 (12.2%)
Full Scale IQ Score	112 (72, 100, 122, 152)
Verbal Comprehension Index (VCI)	110 (65, 100, 120, 140)
Working Memory Index (WMI)	110 (60, 95, 115, 140)
Processing Speed Index (PSI)	110 (55, 100, 125, 145)
Perceptual Reasoning Index (PRI)	110 (65, 95, 125, 130)

Continuous variables are summarized with the sample median (minimum, first quartile, third quartile, maximum). Information was unavailable in some individuals regarding height (N = 32), weight (N = 35), BMI (N = 35), personality (N = 39), smoking (N = 32), drinking (N = 32), physical exercise (N = 32), and sleep quality (N = 33).

### Associations of subject demographic and lifestyle characteristics with IQ score measures

Associations of demographic and lifestyle factors with the five different IQ measures from multivariable analysis are displayed in Table 2 (Full Scale IQ, VCI, WMI) and Table 3 (PSI, PRI), where as previously mentioned p values of 0.005 or lower are considered as statistically significant after adjustment for multiple testing. Older age was significantly associated with a higher score for each IQ measure. Increased height was associated with a higher PRI. Smoking was associated with significantly lower scores for all IQ measures except PSI. Subjects with better sleep quality had a significantly higher score for Full Scale IQ, VCI and WMI. Gender, weight, BMI, personality, alcohol consumption, and physical exercise were not significantly associated with any IQ measures after adjustment for multiple testing. These results were relatively consistent in single variable analysis without adjustment for any confounding variables (S1 and S2 Tables).

**Table 2:** Associations of subject demographic and lifestyle characteristics with IQ score measures (Full Scale IQ Score, VCI, and WMI) from multivariable analysis.

	Association with Full Scale IQ score		Association with VCI		Association with WMI	
Variable	Regression coefficient (95% CI)	P-value	Regression coefficient (95% CI)	P-value	Regression coefficient (95% CI)	P-value
Age (1 year increase)	2.30 (1.74, 2.87)	<0.001	2.59 (2.02, 3.16)	<0.001	2.77 (2.19, 3.34)	<0.001
Gender (Male)	-1.28 (-5.14, 2.59)	0.52	1.03 (-2.91, 4.98)	0.61	1.76 (-2.19, 5.71)	0.38
Height (0.1 m increase)	2.78 (0.53, 5.02)	0.015	2.40 (0.11, 4.68)	0.040	0.93 (-1.37, 3.23)	0.43
Weight (10 kg increase)	1.06 (-0.47, 2.60)	0.17	1.19 (-0.37, 2.75)	0.13	0.90 (-0.66, 2.45)	0.26
BMI (5 unit increase)	0.61 (-1.91, 3.12)	0.63	1.00 (-1.55, 3.56)	0.44	1.34 (-1.21, 3.89)	0.30
Personality (Introvert)	0.10 (-2.47, 2.66)	0.94	-0.71 (-3.32, 1.91)	0.60	-0.12 (-2.75, 2.50)	0.93
Smoking						
No	0.00 (reference)	N/A	0.00 (reference)	N/A	0.00 (reference)	N/A
Yes	-8.61 (-12.05, -5.16)	<0.001	-7.17 (-10.68, -3.66)	<0.001	-8.47 (-11.99, -4.95)	<0.001
Alcohol consumption						
No	0.00 (reference)	N/A	0.00 (reference)	N/A	0.00 (reference)	N/A
Yes	-2.65 (-5.41, 0.11)	0.060	-2.91 (-5.72, -0.10)	0.043	-3.17 (-5.98, -0.35)	0.028
Physical exercise	Test of overall difference: P = 0.008		Test of overall difference: P = 0.012		Test of overall difference: P = 0.023	
Often	0.00 (reference)	N/A	0.00 (reference)	N/A	0.00 (reference)	N/A
Once a week	1.89 (-0.96, 4.75)	0.19	2.40 (-0.51, 5.32)	0.11	2.04 (-0.88, 4.96)	0.17
Once a month	-0.13 (-4.29, 4.03)	0.95	1.60 (-2.64, 5.83)	0.46	1.02 (-3.23, 5.27)	0.64
Rare	-3.66 (-6.95, -0.37)	0.029	-2.85 (-6.20, 0.50)	0.095	-2.95 (-6.31, 0.41)	0.085
Sleep quality	Test of overall difference: P<0.001		Test of overall difference: P<0.001		Test of overall difference: P = 0.004	
High	0.00 (reference)	N/A	0.00 (reference)	N/A	0.00 (reference)	N/A
Intermediate	-5.83 (-8.46, -3.20)	<0.001	-6.52 (-9.20, -3.84)	<0.001	-4.47 (-7.15, -1.78)	0.001
Low	-5.84 (-9.97, -1.71)	0.006	-6.82 (-11.03, -2.60)	0.002	-2.18 (-6.41, 2.04)	0.31

Regression coefficients, 95% CIs, and p values were calculated from multivariable linear regression models. Regression coefficients are interpreted as the change in the mean IQ measure corresponding to the increase specified in parenthesis (continuous variables) or presence of the given characteristic (categorical variables). For all variables except height, weight, and BMI, models were adjusted for age, gender, height, weight, BMI, personality, smoking, alcohol consumption, physical exercise, and sleep quality. For height, weight, and BMI, models were adjusted for age, gender, personality, smoking, alcohol consumption, physical exercise, and sleep quality. P values of 0.005 or lower were considered as statistically significant after applying a Bonferroni correction for multiple testing. CI = confidence interval.

**Table 3:** Associations of subject demographic and lifestyle characteristics with IQ score measures (PSI and PRI) from multivariable analysis.

	Association with PSI		Association with PRI	
Variable	Regression coefficient (95% CI)	P-value	Regression coefficient (95% CI)	P-value
Age (1 year increase)	0.89 (0.29, 1.50)	0.004	0.95 (0.32, 1.59)	0.003
Gender (Male)	-4.54 (-8.71, -0.38)	0.033	-2.33 (-6.69, 2.03)	0.29
Height (0.1 m increase)	1.06 (-1.36, 3.49)	0.39	4.07 (1.53, 6.60)	0.002
Weight (10 kg increase)	-0.82 (-2.46, 0.83)	0.33	2.16 (0.44, 3.89)	0.01
BMI (5 unit increase)	-1.97 (-4.67, 0.72)	0.15	1.87 (-0.97, 4.72)	0.20
Personality (Introvert)	-0.51 (-3.27, 2.25)	0.72	1.59 (-1.30, 4.48)	0.28
Smoking				
No	0.00 (reference)	N/A	0.00 (reference)	N/A
Yes	-4.27 (-7.98, -0.56)	0.024	-6.29 (-10.18, -2.40)	0.002
Alcohol consumption				
No	0.00 (reference)	N/A	0.00 (reference)	N/A
Yes	-0.97 (-3.94, 2.00)	0.52	-1.37 (-4.48, 1.74)	0.39
Physical exercise	Test of overall difference: P = 0.22		Test of overall difference: P = 0.33	
Often	0.00 (reference)	N/A	0.00 (reference)	N/A
Once a week	0.46 (-2.62, 3.54)	0.77	0.95 (-2.27, 4.18)	0.56
Once a month	-1.82 (-6.30, 2.65)	0.42	-0.57 (-5.26, 4.11)	0.81
Rare	-2.97 (-6.50, 0.57)	0.10	-2.41 (-6.12, 1.29)	0.20
Sleep quality	Test of overall difference: P = 0.024		Test of overall difference: P = 0.058	
High	0.00 (reference)	N/A	0.00 (reference)	N/A
Intermediate	-3.48 (-6.31, -0.65)	0.016	-3.47 (-6.44, -0.51)	0.022
Low	-5.08 (-9.53, -0.63)	0.025	-3.86 (-8.52, 0.80)	0.10

Regression coefficients, 95% CIs, and p values were calculated from multivariable linear regression models. Regression coefficients are interpreted as the change in the mean IQ measure corresponding to the increase specified in parenthesis (continuous variables) or presence of the given characteristic (categorical variables). For all variables except height, weight, and BMI, models were adjusted for age, gender, height, weight, BMI, personality, smoking, alcohol consumption, physical exercise, and sleep quality. For height, weight, and BMI, models were adjusted for age, gender, personality, smoking, alcohol consumption, physical exercise, and sleep quality. P values of 0.005 or lower were considered as statistically significant after applying a Bonferroni correction for multiple testing. CI = confidence interval.

### Associations of *APOE* genotype with subject IQ score measures

An evaluation of the association between *APOE* ε4 allele and each of the five different IQ measures was shown in Table 4. In single variable (i.e. unadjusted) analysis, there was no evidence of an association between *APOE* ε4 and Full Scale IQ (P = 0.70), VCI (P = 0.43), WMI (P = 0.44), PSI (P = 0.57), PRI (P = 0.94). This lack of an association between *APOE* ε4 and the different IQ measures was consistent in multivariable analysis which adjusts for the potential confounding influences of school, age, gender, height, weight, BMI, personality, smoking, drinking, physical exercise, and sleep quality (all P≥0.20, Table 4). Similarly, there was no evidence of an association between *APOE* ε2 and any of the IQ measures that were considered, either in single variable analysis (P≥0.091, Table 5) or in multivariable analysis adjusting for the aforementioned eleven factors (P≥0.29, Table 5).

**Table 4:** Associations between *APOE* ε4 and IQ score measures.

			Association between *APOE* ε4 and the given IQ measure			
	Median (Min, Q1, Q3, Max)		Single variable analysis		Multivariable analysis	
IQ measure	ε4 allele not present (N = 488)	ε4 allele present (N = 119)	Regression coefficient (95% CI)	P-value	Regression coefficient (95% CI)	P-value
Full Scale IQ Score	112 (72, 100, 121, 152)	115 (74, 97, 122, 140)	-0.59 (-3.62, 2.45)	0.70	0.59 (-1.53, 2.71)	0.58
Verbal Comprehension Index (VCI)	110 (65, 100, 120, 140)	110 (70, 95, 120, 135)	-1.26 (-4.36, 1.85)	0.43	0.08 (-2.21, 2.37)	0.94
Working Memory Index (WMI)	110 (60, 95, 115, 140)	105 (70, 95, 115, 140)	-1.22 (-4.35, 1.91)	0.44	0.22 (-2.07, 2.51)	0.85
Processing Speed Index (PSI)	110 (55, 100, 120, 145)	110 (80, 100, 125, 145)	0.89 (-2.21, 3.99)	0.57	1.95 (-1.00, 4.90)	0.20
Perceptual Reasoning Index (PRI)	110 (70, 95, 120, 140)	110 (65, 95, 120, 145)	-0.12 (-3.29, 3.05)	0.94	-0.06 (-2.96, 2.84)	0.97

Regression coefficients, 95% CIs, and p values were calculated from linear regression models. Regression coefficients are interpreted as the difference in means of the given IQ measure between carriers and non-carriers of the ε4 allele (i.e. ε4 allele present vs. ε4 allele not present). A regression coefficient greater than “0” indicates a higher value of the given measure for ε4 carriers, and a regression coefficient less than “0” indicates a lower value of the given measure for ε4 carriers. Multivariable models were adjusted for school (Xiamen University or AnFang College), age, gender, height, weight, BMI, personality, smoking, drinking alcohol, physical exercise, and sleep quality. P values of 0.01 or lower were considered as statistically significant after applying a Bonferroni correction for multiple testing. Min = Minimum; Q1 = first quartile; Q3 = third quartile; Max = Maximum; CI = confidence interval.

**Table 5:** Associations between *APOE* ε2 and IQ score measures.

			Association between *APOE* ε4 and the given IQ measure			
	Median (Min, Q1, Q3, Max)		Single variable analysis		Multivariable analysis	
IQ measure	ε2 allele not present (N = 519)	ε2 allele present (N = 88)	Regression coefficient (95% CI)	P-value	Regression coefficient (95% CI)	P-value
Full Scale IQ Score	112 (72, 100, 120, 152)	114 (76, 104, 122, 134)	1.16 (-2.27, 4.58)	0.51	0.03 (-2.41, 2.48)	0.98
Verbal Comprehension Index (VCI)	110 (65, 100, 120, 140)	110 (65, 98, 120, 130)	-0.03 (-3.53, 3.47)	0.99	-0.70 (-3.33, 1.94)	0.60
Working Memory Index (WMI)	110 (60, 95, 115, 140)	110 (65, 100, 115, 140)	0.68 (-2.85, 4.21)	0.70	-0.11 (-2.75, 2.53)	0.93
Processing Speed Index (PSI)	110 (55, 100, 125, 145)	115 (80, 105, 125, 145)	3.00 (-0.48, 6.49)	0.091	1.85 (-1.56, 5.25)	0.29
Perceptual Reasoning Index (PRI)	110 (65, 95, 120, 145)	110 (70, 95, 120, 135)	0.16 (-3.42, 3.73)	0.93	-0.80 (-4.14, 2.53)	0.64

Regression coefficients, 95% CIs, and p values were calculated from linear regression models. Regression coefficients are interpreted as the difference in means of the given IQ measure between carriers and non-carriers of the ε2 allele (i.e. ε2 allele present vs. ε2 allele not present). A regression coefficient greater than “0” indicates a higher value of the given measure for ε2 carriers, and a regression coefficient less than “0” indicates a lower value of the given measure for ε2 carriers. Multivariable models were adjusted for school (Xiamen University or AnFang College), age, gender, height, weight, BMI, personality, smoking, drinking alcohol, physical exercise, and sleep quality. P values of 0.01 or lower were considered as statistically significant after applying a Bonferroni correction for multiple testing. Min = Minimum; Q1 = first quartile; Q3 = third quartile; Max = Maximum; CI = confidence interval.

## Discussion

In this study, we analyzed the associations among *APOE* genotype, demographic and lifestyle characteristics, and intelligence scores in a cohort of 607 Chinese college students aged 18 to 25 years old. We found that neither *APOE* ε4 nor ε2 has significant association with different IQ measures either in single variable or multivariable analysis which adjusts for potentially confounding variables. Interestingly, demographic and lifestyle characteristics, including age, smoking and sleep quality, were significantly associated with different IQ measures.

Human apoE is polymorphic with three common isoforms that have different effects on lipid and neuronal homeostasis. *APOE* ε3 is the most common and ε2 is the least common allele. The *APOE* allele frequency for ε2, ε3 and ε4 in our Chinese Han population was 7.6%, 82.0%, 10.4%, respectively. Thus, the frequency of the ε4 allele was lower than that for the world-wide population (8.4%, 77.9% and 13.7%for ε2, ε3 and ε4, respectively) [12].

Numerous studies have demonstrated that *APOE* ε4 has extensive deleterious effects on several biological processes. The possession of at least one ε4 allele increases three-fold for the risk of AD [30]. Moreover, *APOE* ε4 appears to be less efficient in delivering cholesterol and essential lipids for maintenance of synaptic integrity and plasticity than *APOE* ε2 and ε3 [31, 32]. *APOE* ε4 has also been experimentally related to exacerbated pro-inflammatory and/or reduced anti-inflammatory functions [22, 33]. However, *APOE* ε4 seems to be protective against infectious diseases such as hepatitis C virus and malaria [34, 35]. The effect of the *APOE* ε4 allele on cognitive function in the elder people is well-characterized [9, 10], but the few studies that examined the role of *APOE* ε4 on cognitive abilities in young population reported inconsistent results. Young adults and children carrying the ε4 allele were reported to exhibit better cognitive performance compared to non-ε4 carriers [26, 27, 36]. Equally though, there are studies which found no benefits of the ɛ4 allele for cognition in early adulthood [37–39]. The discrepancy among results from different studies might be attributed to variation in the sample size or ethnic population. Our study in a cohort of 607 young adults of the Chinese Han population revealed no evidence that cognitive performance was related to the *APOE* genotype.

We found evidence of associations between demographic and lifestyle characteristics with different IQ measures. Intriguingly, smoking was found to be associated with significantly lower IQ scores. The relatively small sample size of our studied population would not allow us to distinguish heavy or light smokers according to the amount of cigarette consumption. Nonetheless, our results are consistent with previous report that smoking was associated with impairments in cognitive function [40]. We also observed that students with better sleep quality tend to score higher in Full Scale IQ, VCI and WMI, indicating a positive association between sleep quality and cognitive function as previously reported [41]. Sleep quality has been suggested to impact hippocampal activation and memory performance [42, 43]. There is compelling evidence showing that sleep promotes memory consolidation and brain plasticity [44–46]. The mechanism remains unclear, but may involve local synaptic changes. Therefore, a portion of individual intelligence differences in our studied population might be attributed to their demographic and lifestyle characteristics, including age, smoking and sleep quality. Future extension of these findings is necessary to clarify whether the demographic and lifestyle factors are the primary cause or merely a downstream consequence of different IQ levels.

In summary, the results of our current study indicate that the *APOE* ε4 allele, a major genetic risk factor for AD, is not notably associated with IQ scores in a cohort of 607 Chinese college students aged 18 to 25 years old. Although we cannot exclude the possibility of a small association between *APOE* ε4 and IQ scores given our results, based on 95% confidence limits we can reasonably conclude that a moderate or strong association is unlikely. Similarly, the *APOE* ε2 did not appear to be associated with any of the IQ measures that were examined. However, we did identified several significant associations of demographic and lifestyle characteristics with the different IQ measures. This study partially advances the understanding of individual intelligence differences and might help to ameliorate declines in cognitive function by adjusting lifestyles.

## Supporting Information

S1 Table Associations of subject demographic and lifestyle characteristics with IQ score measures (IQ Full Scale score, VVI, and WMI) from single variable analysis. (DOCX)

S2 Table Associations of subject demographic and lifestyle characteristics with IQ score measures (PSI and PRI)) from single variable analysis. (DOCX)

## References

[1] Deary IJ. Intelligence. Annual review of psychology. 2012;63:453–82. 10.1146/annurev-psych-120710-100353 . 21943169

[2] Deary IJ, Penke L, Johnson W. The neuroscience of human intelligence differences. Nature reviews Neuroscience. 2010;11(3):201–11. 10.1038/nrn2793 . 20145623

[3] Nisbett RE, Aronson J, Blair C, Dickens W, Flynn J, Halpern DF, et al Intelligence: new findings and theoretical developments. The American psychologist. 2012;67(2):130–59. 10.1037/a0026699 . 22233090

[4] Deary IJ, Johnson W, Houlihan LM. Genetic foundations of human intelligence. Human genetics. 2009;126(1):215–32. 10.1007/s00439-009-0655-4 . 19294424

[5] Deary IJ, Strand S, Smith P, Fernandes C. Intelligence and educational achievement. Intelligence. 2007;35(1):13–21. 10.1016/j.intell.2006.02.001 WOS:000243867000002.

[6] Loeber S, Duka T, Welzel H, Nakovics H, Heinz A, Flor H, et al Impairment of Cognitive Abilities and Decision Making after Chronic Use of Alcohol: The Impact of Multiple Detoxifications. Alcohol Alcoholism. 2009;44(4):372–81. 10.1093/alcalc/agp030 WOS:000267440300005. 19487491

[7] Cai SR, Pang WW, Low YL, Sim LW, Sam SC, Bruntraeger MB, et al Infant feeding effects on early neurocognitive development in Asian children. American Journal of Clinical Nutrition. 2015;101(2):326–36. 10.3945/ajcn.114.095414 WOS:000348973900012. 25646330

[8] Giorgetti R, Marcotulli D, Tagliabracci A, Schifano F. Effects of ketamine on psychomotor, sensory and cognitive functions relevant for driving ability. Forensic science international. 2015;252:127–42. 10.1016/j.forsciint.2015.04.024 . 25981945

[9] Caselli RJ, Reiman EM, Locke DE, Hutton ML, Hentz JG, Hoffman-Snyder C, et al Cognitive domain decline in healthy apolipoprotein E epsilon4 homozygotes before the diagnosis of mild cognitive impairment. Archives of neurology. 2007;64(9):1306–11. 10.1001/archneur.64.9.1306 . 17846270

[10] Wisdom NM, Callahan JL, Hawkins KA. The effects of apolipoprotein E on non-impaired cognitive functioning: a meta-analysis. Neurobiology of aging. 2011;32(1):63–74. 10.1016/j.neurobiolaging.2009.02.003 . 19285755

[11] Mahley RW, Rall SC Jr. Apolipoprotein E: far more than a lipid transport protein. Annu Rev Genomics Hum Genet. 2000;1:507–37. 10.1146/annurev.genom.1.1.507 . 11701639

[12] Farrer LA, Cupples LA, Haines JL, Hyman B, Kukull WA, Mayeux R, et al Effects of age, sex, and ethnicity on the association between apolipoprotein E genotype and Alzheimer disease. A meta-analysis. APOE and Alzheimer Disease Meta Analysis Consortium. Jama. 1997;278(16):1349–56. . 9343467

[13] Bu G. Apolipoprotein E and its receptors in Alzheimer's disease: pathways, pathogenesis and therapy. Nature reviews Neuroscience. 2009;10(5):333–44. 10.1038/nrn2620 19339974 PMC2908393

[14] Medway CW, Abdul-Hay S, Mims T, Ma L, Bisceglio G, Zou F, et al ApoE variant p.V236E is associated with markedly reduced risk of Alzheimer's disease. Molecular neurodegeneration. 2014;9:11 10.1186/1750-1326-9-11 24607147 PMC3995879

[15] Corder EH, Saunders AM, Strittmatter WJ, Schmechel DE, Gaskell PC, Small GW, et al Gene dose of apolipoprotein E type 4 allele and the risk of Alzheimer's disease in late onset families. Science. 1993;261(5123):921–3. . 8346443 10.1126/science.8346443

[16] Castellano JM, Kim J, Stewart FR, Jiang H, DeMattos RB, Patterson BW, et al Human apoE isoforms differentially regulate brain amyloid-beta peptide clearance. Sci Transl Med. 2011;3(89):89ra57 10.1126/scitranslmed.3002156 21715678 PMC3192364

[17] Liraz O, Boehm-Cagan A, Michaelson DM. ApoE4 induces Abeta42, tau, and neuronal pathology in the hippocampus of young targeted replacement apoE4 mice. Molecular neurodegeneration. 2013;8:16 10.1186/1750-1326-8-16 23684315 PMC3659080

[18] Tai LM, Mehra S, Shete V, Estus S, Rebeck GW, Bu G, et al Soluble apoE/Abeta complex: mechanism and therapeutic target for APOE4-induced AD risk. Molecular neurodegeneration. 2014;9:2 10.1186/1750-1326-9-2 24386905 PMC3897976

[19] Mahley RW. Apolipoprotein E: cholesterol transport protein with expanding role in cell biology. Science. 1988;240(4852):622–30. . 3283935 10.1126/science.3283935

[20] Ossenkoppele R, van der Flier WM, Zwan MD, Adriaanse SF, Boellaard R, Windhorst AD, et al Differential effect of APOE genotype on amyloid load and glucose metabolism in AD dementia. Neurology. 2013;80(4):359–65. 10.1212/WNL.0b013e31827f0889 . 23255822

[21] Hoe HS, Harris DC, Rebeck GW. Multiple pathways of apolipoprotein E signaling in primary neurons. Journal of neurochemistry. 2005;93(1):145–55. 10.1111/j.1471-4159.2004.03007.x . 15773914

[22] Keene CD, Cudaback E, Li X, Montine KS, Montine TJ. Apolipoprotein E isoforms and regulation of the innate immune response in brain of patients with Alzheimer's disease. Current opinion in neurobiology. 2011;21(6):920–8. 10.1016/j.conb.2011.08.002 21907569 PMC3237894

[23] Chang S, ran Ma T, Miranda RD, Balestra ME, Mahley RW, Huang Y. Lipid- and receptor-binding regions of apolipoprotein E4 fragments act in concert to cause mitochondrial dysfunction and neurotoxicity. Proc Natl Acad Sci U S A. 2005;102(51):18694–9. 10.1073/pnas.0508254102 16344479 PMC1311737

[24] Valla J, Yaari R, Wolf AB, Kusne Y, Beach TG, Roher AE, et al Reduced posterior cingulate mitochondrial activity in expired young adult carriers of the APOE epsilon4 allele, the major late-onset Alzheimer's susceptibility gene. J Alzheimers Dis. 2010;22(1):307–13. 10.3233/JAD-2010-100129 20847408 PMC3124564

[25] Liu CC, Kanekiyo T, Xu H, Bu G. Apolipoprotein E and Alzheimer disease: risk, mechanisms and therapy. Nat Rev Neurol. 2013;9(2):106–18. 10.1038/nrneurol.2012.263 23296339 PMC3726719

[26] Mondadori CR, de Quervain DJ, Buchmann A, Mustovic H, Wollmer MA, Schmidt CF, et al Better memory and neural efficiency in young apolipoprotein E epsilon4 carriers. Cereb Cortex. 2007;17(8):1934–47. 10.1093/cercor/bhl103 . 17077159

[27] Wright RO, Hu H, Silverman EK, Tsaih SW, Schwartz J, Bellinger D, et al Apolipoprotein E genotype predicts 24-month bayley scales infant development score. Pediatr Res. 2003;54(6):819–25. 10.1203/01.PDR.0000090927.53818.DE . 12930912

[28] Bloss CS, Delis DC, Salmon DP, Bondi MW. Decreased cognition in children with risk factors for Alzheimer's disease. Biological psychiatry. 2008;64(10):904–6. 10.1016/j.biopsych.2008.07.004 18722591 PMC2607139

[29] Deary IJ, Whiteman MC, Pattie A, Starr JM, Hayward C, Wright AF, et al Cognitive change and the APOE epsilon 4 allele. Nature. 2002;418(6901):932 10.1038/418932a . 12198535

[30] Saunders AM, Strittmatter WJ, Schmechel D, George-Hyslop PH, Pericak-Vance MA, Joo SH, et al Association of apolipoprotein E allele epsilon 4 with late-onset familial and sporadic Alzheimer's disease. Neurology. 1993;43(8):1467–72. . 8350998 10.1212/wnl.43.8.1467

[31] Rapp A, Gmeiner B, Huttinger M. Implication of apoE isoforms in cholesterol metabolism by primary rat hippocampal neurons and astrocytes. Biochimie. 2006;88(5):473–83. 10.1016/j.biochi.2005.10.007 . 16376010

[32] Hamanaka H, Katoh-Fukui Y, Suzuki K, Kobayashi M, Suzuki R, Motegi Y, et al Altered cholesterol metabolism in human apolipoprotein E4 knock-in mice. Human molecular genetics. 2000;9(3):353–61. . 10655544 10.1093/hmg/9.3.353

[33] Lynch JR, Tang W, Wang H, Vitek MP, Bennett ER, Sullivan PM, et al APOE genotype and an ApoE-mimetic peptide modify the systemic and central nervous system inflammatory response. The Journal of biological chemistry. 2003;278(49):48529–33. 10.1074/jbc.M306923200 . 14507923

[34] Kuhlmann I, Minihane AM, Huebbe P, Nebel A, Rimbach G. Apolipoprotein E genotype and hepatitis C, HIV and herpes simplex disease risk: a literature review. Lipids in health and disease. 2010;9:8 10.1186/1476-511X-9-8 20109174 PMC2830997

[35] Fujioka H, Phelix CF, Friedland RP, Zhu X, Perry EA, Castellani RJ, et al Apolipoprotein E4 prevents growth of malaria at the intraerythrocyte stage: implications for differences in racial susceptibility to Alzheimer's disease. Journal of health care for the poor and underserved. 2013;24(4 Suppl):70–8. 10.1353/hpu.2014.0009 . 24241262 PMC4909051

[36] Schultz MR, Lyons MJ, Franz CE, Grant MD, Boake C, Jacobson KC, et al Apolipoprotein E genotype and memory in the sixth decade of life. Neurology. 2008;70(19 Pt 2):1771–7. 10.1212/01.wnl.0000286941.74372.cc 18235080 PMC3107734

[37] Bunce D, Bielak AA, Anstey KJ, Cherbuin N, Batterham PJ, Easteal S. APOE genotype and cognitive change in young, middle-aged, and older adults living in the community. J Gerontol A Biol Sci Med Sci. 2014;69(4):379–86. 10.1093/gerona/glt103 . 23902936

[38] Taylor AE, Guthrie PA, Smith GD, Golding J, Sattar N, Hingorani AD, et al IQ, educational attainment, memory and plasma lipids: associations with apolipoprotein E genotype in 5995 children. Biological psychiatry. 2011;70(2):152–8. 10.1016/j.biopsych.2010.10.033 21215387 PMC3130925

[39] Turic D, Fisher PJ, Plomin R, Owen MJ. No association between apolipoprotein E polymorphisms and general cognitive ability in children. Neurosci Lett. 2001;299(1–2):97–100. . 11166947 10.1016/s0304-3940(00)01789-4

[40] Richards M, Jarvis MJ, Thompson N, Wadsworth ME. Cigarette smoking and cognitive decline in midlife: evidence from a prospective birth cohort study. Am J Public Health. 2003;93(6):994–8. 12773367 10.2105/ajph.93.6.994 PMC1447882

[41] Benitez A, Gunstad J. Poor sleep quality diminishes cognitive functioning independent of depression and anxiety in healthy young adults. Clin Neuropsychol. 2012;26(2):214–23. 10.1080/13854046.2012.658439 . 22335237

[42] Van Der Werf YD, Altena E, Schoonheim MM, Sanz-Arigita EJ, Vis JC, De Rijke W, et al Sleep benefits subsequent hippocampal functioning. Nature neuroscience. 2009;12(2):122–3. 10.1038/nn.2253 . 19151712

[43] Yoo SS, Hu PT, Gujar N, Jolesz FA, Walker MP. A deficit in the ability to form new human memories without sleep. Nature neuroscience. 2007;10(3):385–92. 10.1038/nn1851 . 17293859

[44] Stickgold R. Sleep-dependent memory consolidation. Nature. 2005;437(7063):1272–8. 10.1038/nature04286 . 16251952

[45] Walker MP, Stickgold R. Sleep-dependent learning and memory consolidation. Neuron. 2004;44(1):121–33. 10.1016/j.neuron.2004.08.031 . 15450165

[46] Maquet P. The role of sleep in learning and memory. Science. 2001;294(5544):1048–52. 10.1126/science.1062856 . 11691982

