# Supplementary material for: Demographic and Lifestyle Characteristics, but Not Apolipoprotein E Genotype, Are Associated with Intelligence among Young Chinese College Students
Source: PLoS One. 2015 Nov 17;10(11):e0143157. doi: 10.1371/journal.pone.0143157 (PMC4648581; doi:10.1371/journal.pone.0143157)
Supplement: S1 Table — (DOCX) [file pone.0143157.s001.docx]

**S1 Table: Associations of subject demographic and lifestyle characteristics with IQ score measures (IQ Full Scale score, VVI, and WMI) from single variable analysis**

|  | Association with IQ Full Scale score | | Association with VCI | | Association with WMI | |
| --- | --- | --- | --- | --- | --- | --- |
| Variable | Regression coefficient (95% CI) | P-value | Regression coefficient (95% CI) | P-value | Regression coefficient (95% CI) | P-value |
| Age (1 year increase) | 2.28 (1.72, 2.83) | <0.001 | 2.67 (2.12, 3.23) | <0.001 | 2.69 (2.13, 3.26) | <0.001 |
| Gender (Male) | -1.63 (-4.06, 0.80) | 0.19 | 0.67 (-1.81, 3.16) | 0.60 | -1.14 (-3.65, 1.37) | 0.37 |
| Height (0.1 m increase) | 0.09 (-1.51, 1.70) | 0.91 | 1.11 (-0.53, 2.76) | 0.18 | -0.39 (-2.04, 1.26) | 0.64 |
| Weight (10 kg increase) | 0.34 (-0.98, 1.66) | 0.62 | 1.18 (-0.17, 2.53) | 0.087 | 0.41 (-0.95, 1.77) | 0.55 |
| BMI (5 unit increase) | 0.94 (-1.71, 3.58) | 0.49 | 1.94 (-0.77, 4.65) | 0.16 | 1.69 (-1.02, 4.41) | 0.22 |
| Personality (Introvert) | -1.13 (-3.82, 1.56) | 0.41 | -2.02 (-4.78, 0.73) | 0.15 | -0.71 (-3.48, 2.05) | 0.61 |
| Smoking |  |  |  |  |  |  |
| No | 0.00 (reference) | N/A | 0.00 (reference) | N/A | 0.00 (reference) | N/A |
| Yes | -9.19 (-12.35, -6.03) | <0.001 | -7.11 (-10.38, -3.83) | <0.001 | -9.36 (-12.61, -6.11) | <0.001 |
| Alcohol consumption |  |  |  |  |  |  |
| No | 0.00 (reference) | N/A | 0.00 (reference) | N/A | 0.00 (reference) | N/A |
| Yes | -4.22 (-7.00, -1.43) | 0.003 | -3.32 (-6.18, -0.46) | 0.023 | -4.42 (-7.28, -1.57) | 0.003 |
| Physical exercise | Test of overall difference: P=0.016 | | Test of overall difference: P=0.005 | | Test of overall difference: P=0.030 | |
| Often | 0.00 (reference) | N/A | 0.00 (reference) | N/A | 0.00 (reference) | N/A |
| Once a week | 1.84 (-1.25, 4.92) | 0.24 | 1.94 (-1.21, 5.09) | 0.23 | 2.19 (-0.99, 5.36) | 0.18 |
| Once a month | 1.49 (-2.96, 5.95) | 0.51 | 2.51 (-2.04, 7.06) | 0.28 | 2.78 (-1.81, 7.36) | 0.23 |
| Rare | -3.42 (-6.74, -0.10) | 0.044 | -3.87 (-7.27, -0.48) | 0.026 | -2.59 (-6.01, 0.83) | 0.14 |
| Sleep quality | Test of overall difference: P<0.001 | | Test of overall difference: P<0.001 | | Test of overall difference: P=0.002 | |
| High | 0.00 (reference) | N/A | 0.00 (reference) | N/A | 0.00 (reference) | N/A |
| Intermediate | -6.72 (-9.55, -3.89) | <0.001 | -7.50 (-10.39, -4.60) | <0.001 | -5.27 (-8.22, -2.32) | 0.001 |
| Low | -8.41 (-12.64, -4.18) | 0.001 | -8.90 (-13.22, -4.57) | <0.001 | -4.79 (-9.19, -0.39) | 0.033 |

Regression coefficients, 95% CIs, and p values were calculated from single variable (i.e. unadjusted) linear regression models. P values of 0.005 or lower were considered as statistically significant after applying a Bonferroni correction for multiple testing. CI=confidence interval.
