# Supplementary material for: Demographic and Lifestyle Characteristics, but Not Apolipoprotein E Genotype, Are Associated with Intelligence among Young Chinese College Students
Source: PLoS One. 2015 Nov 17;10(11):e0143157. doi: 10.1371/journal.pone.0143157 (PMC4648581; doi:10.1371/journal.pone.0143157)
Supplement: S2 Table — (DOCX) [file pone.0143157.s002.docx]

**S2 Table: Associations of subject demographic and lifestyle characteristics with IQ score measures (PSI and PRI) from single variable analysis**

|  | Association with PSI | | Association with PRI | |
| --- | --- | --- | --- | --- |
| Variable | Regression coefficient (95% CI) | P-value | Regression coefficient (95% CI) | P-value |
| Age (1 year increase) | 0.88 (0.29, 1.47) | 0.004 | 0.88 (0.28, 1.49) | 0.004 |
| Gender (Male) | -5.37 (-7.82, -2.93) | <0.001 | 0.62 (-1.92, 3.16) | 0.63 |
| Height (0.1 m increase) | -2.23 (-3.82, -0.63) | 0.006 | 1.65 (-0.01, 3.31) | 0.051 |
| Weight (10 kg increase) | -2.08 (-3.39, -0.77) | 0.002 | 1.55 (0.18, 2.91) | 0.026 |
| BMI (5 unit increase) | -2.83 (-5.47, -0.20) | 0.035 | 2.34 (-0.39, 5.08) | 0.093 |
| Personality (Introvert) | -1.38 (-4.07, 1.30) | 0.31 | 0.65 (-2.15, 3.45) | 0.65 |
| Smoking |  |  |  |  |
| No | 0.00 (reference) | N/A | 0.00 (reference) | N/A |
| Yes | -6.87 (-10.07, -3.67) | <0.001 | -5.01 (-8.35, -1.66) | 0.003 |
| Alcohol consumption |  |  |  |  |
| No | 0.00 (reference) | N/A | 0.00 (reference) | N/A |
| Yes | -3.37 (-6.16, -0.58) | 0.018 | -2.09 (-4.99, 0.82) | 0.16 |
| Physical exercise | Test of overall difference: P=0.61 | | Test of overall difference: P=0.30 | |
| Often | 0.00 (reference) | N/A | 0.00 (reference) | N/A |
| Once a week | 0.97 (-2.14, 4.07) | 0.54 | 0.63 (-2.59, 3.85) | 0.70 |
| Once a month | -0.20 (-4.69, 4.28) | 0.93 | 0.17 (-4.48, 4.82) | 0.94 |
| Rare | -1.35 (-4.7, 2.00) | 0.43 | -2.60 (-6.07, 0.87) | 0.14 |
| Sleep quality | Test of overall difference: P=0.003 | | Test of overall difference: P=0.012 | |
| High | 0.00 (reference) | N/A | 0.00 (reference) | N/A |
| Intermediate | -3.81 (-6.67, -0.95) | 0.009 | -4.14 (-7.12, -1.15) | 0.007 |
| Low | -6.83 (-11.11, -2.55) | 0.002 | -5.33 (-9.79, -0.87) | 0.019 |

Regression coefficients, 95% CIs, and p values were calculated from single variable (i.e. unadjusted) linear regression models. P values of 0.005 or lower were considered as statistically significant after applying a Bonferroni correction for multiple testing. CI=confidence interval.
